# Rectal–vaginal pressure gradient in patients with pelvic organ prolapse and symptomatic rectocele

**DOI:** 10.1186/s12905-021-01304-6

**Published:** 2021-04-20

**Authors:** Cheng Tan, Man Tan, Jing Geng, Jun Tang, Xin Yang

**Affiliations:** 1 Department of Obstetrics and Gyencology, Peking University People’s Hospital, No. 11 of Xizhimen south Street, Xicheng District, Beijing, 100048 China; 2 Beijing Key Laboratory of Female Pelvic Floor Disorders, Beijing, China

**Keywords:** Rectocele, Obstructed defecation, Rectal pressure, Vaginal pressure, Pelvic organ prolapse

## Abstract

**Objective:**

The aim of this study is to examine the relationship between rectal–vaginal pressure and symptomatic rectocele in patients with pelvic organ prolapse (POP).

**Method:**

Patients with posterior vaginal prolapse staged III or IV in accordance with the POP Quantitation classification method who were scheduled for pelvic floor reconstructive surgery in the years 2016–2019 were included in the study. Rectocele was diagnosed using translabial ultrasound, and obstructed defecation (OD) was diagnosed in accordance with the Roma IV diagnostic criteria. Both rectal and vaginal pressure were measured using peritron manometers at maximum Vasalva. To ensure stability, the test was performed three times with each patient.

**Results:**

A total of 217 patients were enrolled in this study. True rectocele was diagnosed in 68 patients at a main rectal ampulla depth of 19 mm. Furthermore, 36 patients were diagnosed with OD. Symptomatic rectocele was significantly associated with older age (*p* < 0.01), a higher OD symptom score (*p* < 0.001), and a lower grade of apical prolapse (*p* < 0.001). The rectal–vaginal pressure gradient was higher in patients with symptomatic rectocele (37.4 ± 11.7 cm H_2_O) compared with patients with asymptomatic rectocele (16.9 ± 8.4 cm H_2_O, *p* < 0.001), and patients without rectocele (17.1 ± 9.2 cm H_2_O, *p* < 0.001).

**Conclusion:**

The rectal–vaginal pressure gradient was found to be a risk factor for symptomatic rectocele in patients with POP. A rectal–vaginal pressure gradient of > 27.5 cm H_2_O was suggested as the cut-off point of the elevated pressure gradient.

## Introduction

Posterior vaginal prolapse (PVP) is a common condition in patients with pelvic organ prolapse (POP). PVP is related to several anatomical abnormalities, such as rectocele, enterocele, and intussusception. Rectocele is defined as a hernial sac of the anterior rectal wall into the lumen of the vagina [1]. Although rectocele is associated with obstructed defecation (OD), some patients affected by it remain asymptomatic [2]. As functional constipation has many subtypes besides OD, such as slow-transit constipation and constipation-predominant irritable bowel syndrome, clinicians find it difficult to determine the causal relationship between rectocele and constipation and are thereby unable to guarantee symptom relief after treatment [3]. A better understanding of the pathogenesis of symptomatic rectocele could help in patient selection, thus enabling post-surgery symptom relief.

In the present study, the straining bulbocavernosus and straining puborectalis reflexes were evoked during straining in order to make the muscles contract, generating pressure against the high rectal pressure [4, 5]. Weakening of the puborectalis and bulbocavernosus musculatures leads to an increased rectal–vaginal pressure gradient and is considered the pathological basis of rectocele [6]. The rectal–vaginal pressure gradient could be the force that pushes feces into the rectocele hernial sac [7]. Apical support impairment and levator ani muscle injury may also contribute to rectocele development [8]. If the rectal–vaginal pressure gradient could be quantified, it could be used as an objective indicator in clarifying the difference between symptomatic and asymptomatic patients. A peritron manometer is a widely used method for measuring rectal and vaginal pressure. In this study, the use of the peritron manometer was chosen because of its simplicity and reliability when measuring relative pressure [9].

The present study’s hypothesis is that a wide rectal–vaginal pressure gradient could be the pathological basis of symptomatic rectocele. The study’s objective is to examine the relationship between rectal–vaginal pressure and symptomatic rectocele in patients with POP.

## Method

The present study, which was designed as cross-sectional, was approved by the ethics committee of Peking University People’s Hospital. The study included patients with PVP staged III and IV in accordance with the POP Quantitation (POP-Q) classification method who were scheduled for pelvic floor reconstructive surgery in the years 2016–2019 [10]. The exclusion criteria were: (1) slow-transit constipation diagnosed by colon transit study; (2) history of inflammatory bowel disease and anal fissure; (3) history of previous POP or rectum-anal surgery; and (4) inability to participate in the test.

Before the surgery, all subjects in the study underwent standard evaluation procedures, including demographic data collection, medical history investigation, questionnaire assessment, physical examination, and the translabial ultrasound test (TLUS). The OD symptom score of the Altomare questionnaire was employed, and OD was diagnosed in accordance with the Roma IV diagnosis criteria [11, 12]. The OD symptom score referred to the total score of the Altomare OD symptom questionnaire, which is a validated eight-item questionnaire used to access the severity of OD symptoms. A higher OD symptom score referred to more serious OD symptoms. True rectocele was diagnosed using the TLUS (GE Healthcare Voluson E10) and defined as the presence of a discontinuity in the anterior contour of the internal anal sphincter and anterior anorectal muscularis, resulting in a diverticulum of the ampulla. This is indicative of a defect of the recto-vaginal septum [13–15].

The patients in the present study were divided into three groups according to their true rectocele and OD diagnoses: group 1, group 2, and group 3. Group 1 included patients with true rectocele and OD, which is referred to as symptomatic rectocele in this study; group 2 included patients with true rectocele, but without OD; and group 3 included patients without true rectocele.

Rectal and vaginal pressure measurements were performed by an investigator who was blinded to the patient's clinical records [6]. The patients were placed in the lithotomy position. Fasting was not required before the test. The patients' rectums were emptied by defecation or saline enema, and the patients’ rectal pressure was measured using an infinitely compliant balloon attached to a 10–12 Fr catheter. The balloon was introduced via the anus and inserted 10 cm into the rectum. The pressure within the balloon was measured with a peritron manometer (Fuzhou RENXIN Medical Tech Co., Ltd), which then uploaded a data-file for further analysis. Intravaginal pressure was measured with a similar manometer catheter introduced into the vagina at 3–4 cm from the introitus and connected to a pressure meter. The pressure difference was measured at rest and at maximum Vasalva. To ensure stability, the test was performed three times with each patient and the average readings were included in the analysis.

Statistical analysis was carried out using the Statistical Package for Social Sciences v12 (IBM Corp., Armonk, NY, USA) and the Statistical Analysis Software (SAS) v9.3 (Cary CR: SAS institute INC., USA) for PC. Univariate and multivariate logistic regression analyses were employed to predict OD symptoms. The enumeration data were tested for normal distribution using the Kolmogorov–Smirnov analysis, and all enumeration data were compared using the Wilcoxon–Mann–Whitney test (OD symptom score), the rank sum test (parity), or the *t*-test (other enumeration data). Candidate variables that had a univariate analysis p value of < 0.1 or were considered clinically relevant were included in a multivariable model. A *p* value of < 0.05 was considered statistically significant. A Receiver Operating Characteristic (ROC) curve was used for testing the prognostic value of the rectal–vaginal pressure gradient in the prediction of symptomatic rectocele.

## Results

**Fig. 1:**
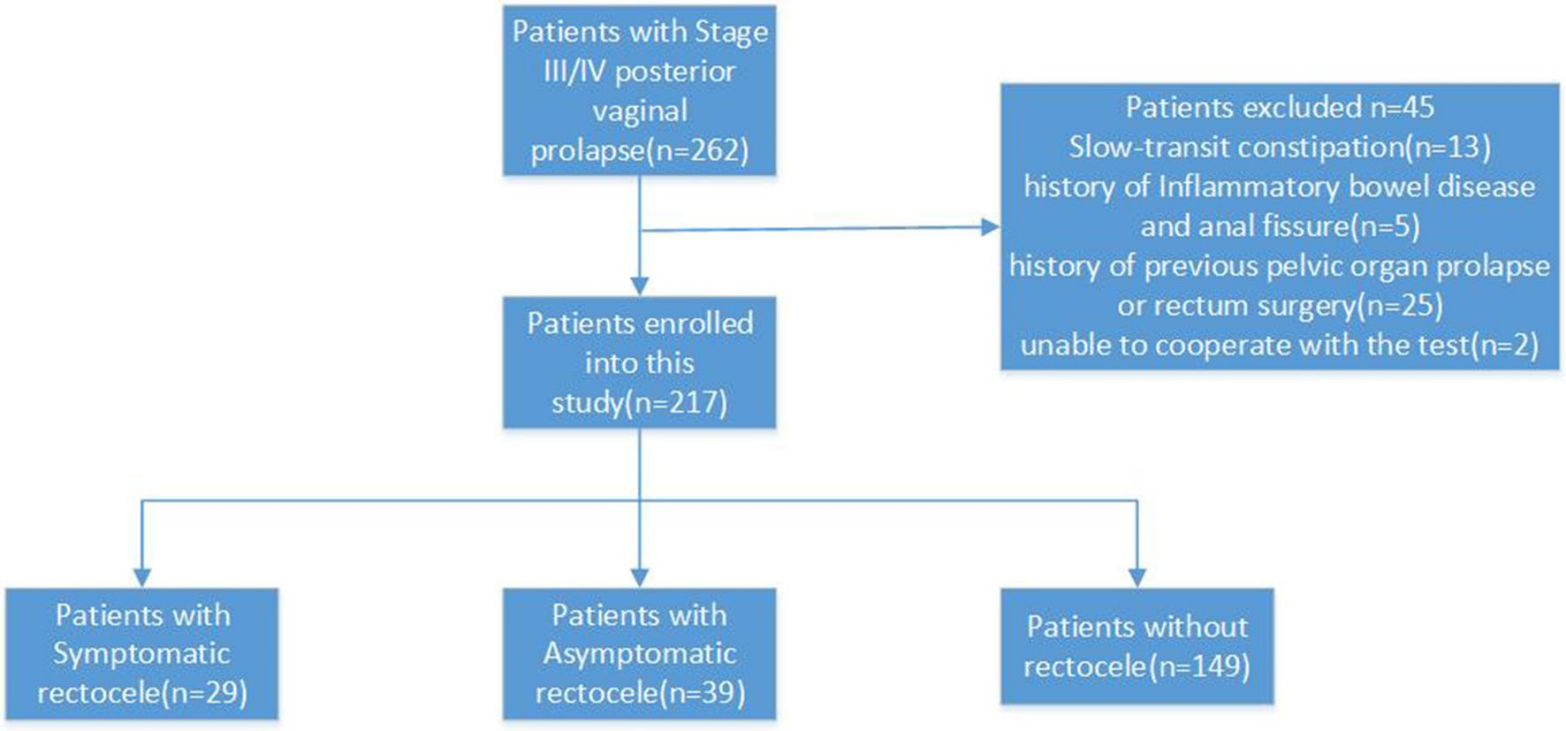
Receiver operating characteristic (ROC) curve for the association between symptomatic rectocele and rectal-vaginal pressure gradient (n = 217). The illustrated cut-off pressure of 27.5 cm H_2_O gives sensitivity of 72.4% and specificity of 93.1%

A total of 262 patients met the inclusion criteria for this study, and 45 patients were excluded for various reasons (Fig. 1). All patients were of Asian ethnicity, and the mean age of participating subjects was 57.5, with a standard deviation (SD) of ± 9.6 years, and a mean body mass index (BMI) of 25.5 (SD ± 4.3) kg/m^2^. The median parity was 2 (ranging from 0 to 6). Ten (4.6%) patients had a history of forceps delivery, and 16 (7.4%) patients had a history of cesarean section.

Moreover, 19 (8.7%) and 5 (2.3%) patients had previously undergone a hysterectomy due to benign gynecological diseases or surgery due to urinary incontinence. None of the patients had a history of obstetrical anal sphincter injury.

All patients were diagnosed with stage III or a higher degree of prolapse in the posterior compartment in accordance with the POP-Q classification method. The patient POP-Q classification is listed in Table 1.

**Table 1:** Patients pelvic organ prolapse according to POP-qualification (n = 217)

	Anterior vaginal prolapse	Apical prolapse	Posterior vaginal prolapse
I	23 (10.6%)	31 (14.3%)	–
II	71 (32.7%)	69 (31.8%)	–
III	102 (41.0%)	98 (45.2%)	190 (87.6%)
IV	21 (9.7%)	19 (8.8%)	27 (12.4%)

TLUS tests were performed on all patients. True rectocele was diagnosed in 68 (31.3%) patients, with a main rectal ampulla depth of 19 mm. Moreover, 4 (1.8%) patients were diagnosed with enterocele (1 and 3 patients in group 2 and group 3, respectively). Thirty-six (16.6%) patients were diagnosed with OD, and their OD symptom scores were significantly higher than in patients without OD (*p* < 0.001). A comparison between the three groups is shown in Table 2. Patients with symptomatic rectocele had a significantly higher age (60.2 ± 9.19 vs. 54.9 ± 9.35, *p* < 0.01) and OD symptom score (8.96 ± 2.83 vs. 1.99 ± 1.05, *p* < 0.001) and a lower apical vaginal prolapse score (27.6% vs. 64.4%, *p* < 0.001) compared with patients without rectocele.

**Table 2:** Single factor analysis comparison among symptomatic rectocele (Group 1 n = 29), asymptomatic rectocele (Group 2 n = 39) and without rectocele (Group 3 n = 149)

	Symptomatic rectocele	Asymptomatic rectocele	Without rectocele
Age (year, mean ± SD)	60.2 ± 9.19	59.3 ± 8.39	54.9 ± 9.35^※^
Menupose (n, %)	22 (75.9%)	31 (79.5%)	103 (69.1%)^※^
BMI (kg/m^2^, mean ± SD)	25.2 ± 2.25	26.1 ± 5.04	25.5 ± 4.47
Parity (mid, min–max)	2 (1–4)	2 (1–6)	2 (0–4)
Forceps delivery (n, %)	2 (6.9%)	2 (5.1%)	6 (4.0%)
History of hysterectomy (n, %)	5 (17.2%)	9 (23.1%)	18 (12.1%)
Cesarean section (n, %)	2 (6.9%)	5 (12.8%)	9 (6.0%)
Stage III/IV anterior prolapse (n, %)	13 (44.8%)	19 (48.7%)	85 (57.0%)
Stage III/IV apical prolapse (n, %)	8 (27.6%)	19 (48.7%)	96 (64.4%)^φ^
Ampulla depth (cm, mean ± SD)	2.3 ± 0.5	1.6 ± 0.3	/
ODS score (mean ± SD)	8.96 ± 2.83^§^	2.46 ± 0.82	1.99 ± 1.05
Vaginal pressure (mean ± SD)	43.4 ± 2.2^§^	66.0 ± 8.36	71.5 ± 9.20
Rectal pressure (mean ± SD)	80.7 ± 9.3	82.0 ± 10.0	88.5 ± 10.9
Rectal–vaginal pressure interval (mean ± SD)	37.2 ± 15.3^§^	16.9 ± 7.4	17.0 ± 7.7

^※^Group 3 showed significant difference compare to Group 1 and Group 2, *p* < 0.01

^φ^Stage of apical prolapse showed significant difference among three group, *p* < 0.001

^§^Group 1 showed significant difference compare to Group 2 and Group 3, *p* < 0.001

**Fig. 2:**
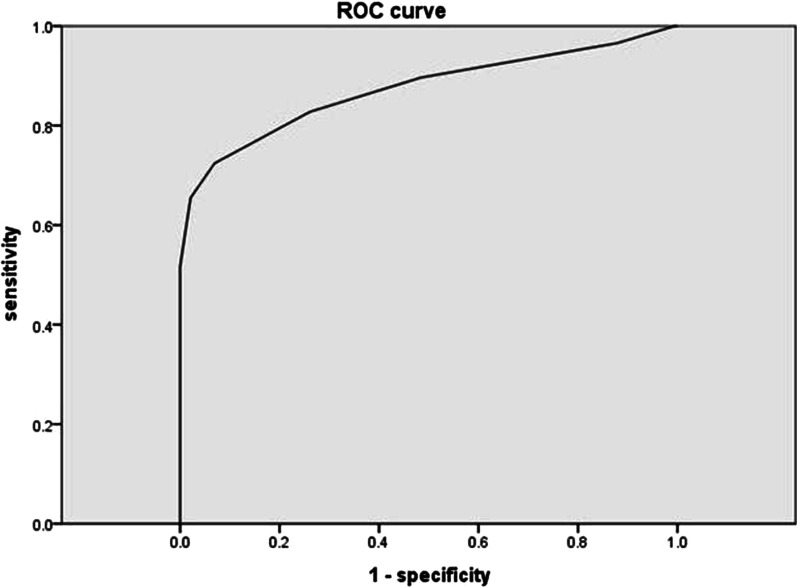
Result from the pressure measurement of among symptomatic rectocele (Group 1 n = 29), asymptomatic rectocele (Group 2 n = 39) and without rectocele (Group 3 n = 149)

**Fig. 3:**
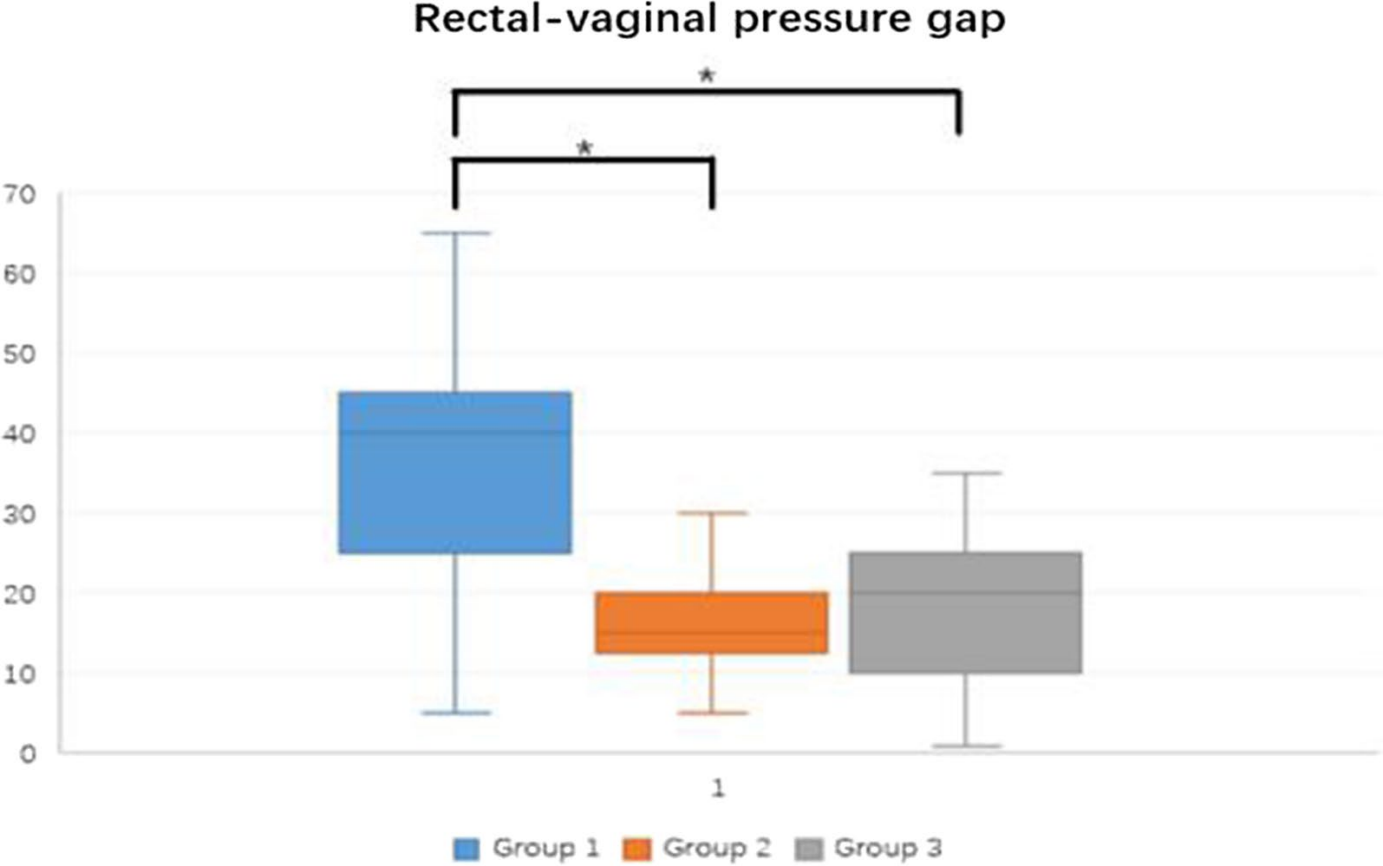
Flow chart

Discomfort was reported by 15 patients during the measurement of rectal and vaginal pressure, and a saline enema was only needed in 6 patients. No other complications or operation delays were found during the test. The rectal–vaginal pressure gradient was higher in patients with symptomatic rectocele (37.2 ± 15.3 cm H_2_O) compared with patients with asymptomatic rectocele (16.9 ± 7.4 cm H_2_O, *p* < 0.001) and patients without rectocele (17.0 ± 7.7 cm H_2_O, *p* < 0.001) (Fig. 2). An ROC curve was used to identify the cut-off point of the pressure gradient that showed the best diagnostic accuracy for symptomatic rectocele in patients with POP. The area-under-the-curve was 0.873 among patients with POP (Fig. 3). An ROC curve analysis also established a pressure gradient of 27.5 cm H_2_O as the most relevant cut-off point for the prediction of symptomatic rectocele (sensitivity 72.4%, specificity 93.1%, positive predictive value 61.8%, and negative predictive value 95.6%). Binary logistic regression analysis showed that the age (OR 1.1), pressure gradient (OR 27.9), and apical prolapse (OR 0.161) were factors associated with symptomatic rectocele (Table 3).

**Table 3:** Logistic regression analysis regarding the risk factors of symptomatic rectocele

	B	*p* value	OR	95% Cl	
				Lower	Upper
Age	0.110	0.031	1.116	1.010	1.233
Elevated pressure interval^a^	3.330	0.001	27.932	9.467	82.411
Severe apical prolapse^b^	− 1.827	0.028	0.161	0.032	0.822

^a^Elevated pressure interval refered to the rectal-vaginal pressure interval > 27.5 cm H_2_O compared with < 27.5 cm H_2_O

^b^Severe apical prolapsed referred to stage III/IV apical prolapse according to POP-Q classification

## Discussion

Rectocele is a common condition in patients with POP, with a variant prevalence rate of 7–50% [16–19]. Among patients with grade II and IV PVP, the prevalence rate of rectocele is 24.5% [20]. Gynecologists tend to diagnose PVP as rectocele; however, only 31.3% patients were diagnosed with true rectocele in this study.

In the present study, it was found that the rectal–vaginal pressure gradient was significantly elevated in patients with symptomatic rectocele, and a cut-off value of > 27.5 cm H_2_O showed good sensitivity and specificity, especially when applied as an exclusion test. The negative predictive value was 95.6%. The aim of this study was to link OD symptoms with an objective indicator with a fair consistency. Pressure gradient measurement could be used as a scanning test for patients with PVP due to its negative predictive value. If a patient has OD symptoms with a normal pressure gradient (< 27.5 cm H_2_O), other possible causes, such as a constipated type of irritable bowel syndrome or slow transit constipation, should be considered.

Although rectocele is considered a major factor in OD development, it is by no means conclusive that rectocele needs to be the cause of OD. There is a lack of objective indicators needed to distinguish symptomatic patients from asymptomatic patients, making the findings of this study rather critical. A study by Dietz demonstrated that a rectal ampulla depth of ≥ 15 mm is associated with OD. Nonetheless, the sensitivities concerning rectocele depth in the prediction of OD symptoms are only 52–66%, limiting the utilization of such methods [21]. A study by Carter found no correlation between symptoms of OD and rectocele size [22]. The study indicated that the rectal–vaginal pressure gradient may be used as an objective factor in POP assessment. Furthermore, this examination can be performed in a consulting room with a portable device. It is safe and easily accepted by patients. Hopefully, this examination will be used as an effective indicator in the prediction of symptomatic relief after surgery in future studies. A recent study found that vaginal pressure is elevated to the same level as rectal pressure with the improvement of OD symptoms after stapled transvaginal rectal resection [23]. This also implies a promising prospect for the method presented in the present study. In addition, novel treatment of OD targeting the rectal–vaginal pressure gradient may be established. The use of a vaginal stent, which is designed to apply pressure on the posterior vaginal wall, leads to OD symptom relief [24].

Another finding of this study was that apical prolapse was independently associated with both symptomatic rectocele and the rectal–vaginal pressure gradient. This finding suggests that apical prolapse could alleviate OD symptoms by reducing the rectal–vaginal pressure gradient. Due to the fact that the vaginal apex and rectum share the same passage of extrusion, one theory is that, in patients with apical prolapse, the abdominal pressure during Vasalva may be directly applied to the anterior wall of the rectum, thus reducing the pressure gradient and OD. It is possible that there is a certain level of bias, as all the patients in this study were from outpatient facilities rather than from communities; although, this study did recruit patients with severe PVP. Further study of the relationship between true rectocele and apical prolapse is necessary.

Although rectal pressure measurement is widely studied, assessing vaginal pressure is still a debated subject in the medical literature due to the complexity of the para-vaginal structure and a great variance in locations [25, 26]. According to recent studies, the segment of the vagina located above the pelvic diaphragm will reflect intra-abdominal pressure, the segment located in the pelvic diaphragm hiatus will measure the squeeze pressure of the pelvic floor muscle, and the vaginal segment located below the diaphragm will reflect atmospheric pressure [27]. A 3D, high-spatial-resolution pressure device, and a vaginal-pressure profile (pull-through) have confirmed that the high-pressure zone of the vagina is located 3–5 cm from the introitus [25, 27]. Therefore, the vaginal balloon was placed 3–5 cm from the introitus. Further study is needed to verify which part of the vagina is most closely related to OD symptoms.

The 3D, high-spatial-resolution pressure device and the infusion vaginal-pressure profile have both been well studied in recent years [27, 28]. However, the peritron manometer was chosen to measure vaginal pressure in this test because it is widely used, can be utilized in the consulting room, and is easily accepted by patients. Because of these features and a high negative predictive value, we suggest a rectal–vaginal pressure test for patients with stage III and IV PVP.

The merits of this study can be found in the adequate sample size, reliable diagnosis method, and blind method application. However, the study has several limitations: (1) anal sphincter function is considered relevant when associated with rectocele and OD, but in this study, there was a lack of sphincter assessment [25]; (2) the examiner had to hold the end of the balloon in almost half of the examinations to prevent it from sliding out of the vagina (it is possible that this is due to artificial interference), and a stent system was designed to reduce the interference and enable further study; (3) the confidence intervals were wide due to a relatively small sample size; and (4) all the patients had POP and were of Asian ethnicity. The results of this study may not necessarily apply to other ethnic groups.

## Conclusion

The rectal–vaginal pressure gradient was found to be a risk factor for symptomatic rectocele in patients with POP. A rectal–vaginal pressure gradient of > 27.5 cm H_2_O was suggested to be the cut-off point of the elevated pressure gradient.

## Data Availability

The datesets used or analyzed during the current study are available from the corresponding author on reasonable request.

## Abbreviations

POP: Pelvic organ prolapse
OD: Obstructed defecation
PVP: Posterior vaginal prolapse
POP-Q: POP quantitation
TLUS: Translabial ultrasound
ROC: Receiver operating characteristic
SD: Standard deviation
BMI: Body mass index
